# Baby’s Online Live Database: An Open Platform for Developmental Science

**DOI:** 10.3389/fpsyg.2021.729302

**Published:** 2021-10-13

**Authors:** Masaharu Kato, Hirokazu Doi, Xianwei Meng, Taro Murakami, Sachiyo Kajikawa, Takashi Otani, Shoji Itakura

**Affiliations:** ^1^Center for Baby Science, Doshisha University, Kyoto, Japan; ^2^Live Database Working Group, Japan Society of Baby Science, Tokyo, Japan; ^3^School of Science and Engineering, Kokushikan University, Tokyo, Japan; ^4^Graduate School of Human Sciences, Osaka University, Suita, Japan; ^5^Department of Education and Psychology, Kyushu Women’s University, Kitakyushu, Japan; ^6^College of Arts and Sciences, Tamagawa University, Machida, Japan; ^7^Department of Psychology, Faculty of Health Science, Kyoto Koka Women’s University, Kyoto, Japan

**Keywords:** COVID-19, longitudinal study, reproducibility, developmental science, open science, survey at home, online study

## Abstract

Efficient data collection in developmental studies is facing challenges due to the decreased birth rates in many regions, reproducibility problems in psychology research, and the COVID-19 pandemic. Here, we propose a novel platform for online developmental science research, the Baby’s Online Live Database (BOLD), which extends the scope of the accessible participant pool, simplifies its management, and enables participant recruitment for longitudinal studies. Through BOLD, researchers can conduct online recruitment of participants preregistered to BOLD simply by specifying their attributes, such as gender and age, and direct the participants to dedicated webpages for each study. Moreover, BOLD handles participant recruitment and reward payment, thereby freeing researchers from the labor of participant management. BOLD also allows researchers the opportunity to access data that were collected from participants in previous research studies. This enables researchers to carry out longitudinal analyses at a relatively low cost. To make BOLD widely accessible, a consortium was formed within the Japan Society of Baby Science, where members from diverse research groups discussed the blueprint of this system. Once in full-scaled operation, BOLD is expected to serve as a platform for various types of online studies and facilitate international collaboration among developmental scientists in the near future.

## Introduction

Developmental science investigates the principles of human beings’ physical and mental abilities from the perspective of development. In this field of research, babies, children, and their caregivers are recruited for observations, surveys, and experiments.

Currently, developmental science faces three major challenges. First, the population of young people who could be participants in developmental science research is decreasing. The birth rate has decreased in many countries; the average fertility rate of the 37 of organisation for economic cooperation and development (OECD) member countries began declining in 1970 and has been hovering below 2.0 since 1991 (OECD, 2021a). The ratio of people under 15years old to the total population in OECD countries has diminished from 25.3% in 1980 to 17.7% in 2018 (OECD, 2021b).

The second challenge is reproducibility. In recent years, the standards for publishing experimental research have become stricter in response to the so-called reproducibility problem (Open Science Collaboration, 2015). The reproducibility crisis showed that good, reliable research often requires larger samples. However, researchers in small- and medium-sized laboratories, who do not have sufficient resources, may struggle to achieve this goal. This situation has a particularly negative impact on the career development of newly independent young principal investigators (PIs) and may lead to a shrinking base in developmental science in the future, and ultimately, the decline of the field.

Finally, the ongoing COVID-19 pandemic has highlighted the vulnerability of human experimental research to the unexpected occurrence of public health concerns and natural disasters. Thus, it is desirable to create infrastructure that enables researchers to continue experimental research in an adverse environment.

Creating a platform where experimental research can be conducted online is a promising solution to the issues listed above, as it would allow for research participation without visiting laboratories. Emerging online tools for experimental research, such as the programming libraries of jsPsych (de Leeuw, 2015), PsyToolkit (Stoet, 2010), and Gorilla (Anwyl-Irvine et al., 2020), and cloud-based sourcing platforms such as Amazon Mechanical Turk, have been of great help to researchers when building and deploying their experiments online. Several systems for conducting online experiments have been proposed to use instead of face-to-face experiments (Frank et al., 2017; Scott and Schulz, 2017; Sheskin and Keil, 2018; Mehr et al., 2019; Rhodes et al., 2020). However, while these systems should contribute to solving the challenges noted above, they are insufficient for overcoming these problems, as they do not help to recruit or manage participants, which is what young PIs and researchers in small- and medium-sized laboratories need. In this paper, we propose the Baby’s Online Live Database (BOLD), an umbrella database system suitable for participant recruitment and management. Sheskin et al. (2020) have previously emphasized the necessity of such an online platform, and our platform may be the first to be implemented. The main aim of BOLD is to provide a large-scale (e.g., national) participant database that can be widely used to run experiments in developmental science.

Additionally, BOLD aims to enhance collaborative and longitudinal studies. Using BOLD, researchers can gain access to participants’ task history, including detailed information on studies that the participants have completed, and their performance. This allows researchers to both link participants’ performance across studies for comparison and perform longitudinal analyses. To achieve this, the participants would need to be engaged in BOLD long-term. Thus, the importance of including research topics that interest and motivate participants to join our database and stay involved is emphasized.

In this perspective paper, we describe the blueprint for BOLD. Implementation is ongoing, and full-scale operation is expected to begin in late summer 2021. Our goal is to make BOLD available to everyone interested in developmental research. Therefore, a working group was formed within the Japan Society of Baby Science (JSBS), in which the core members of the working group, who come from diverse research groups, discuss the basic design of BOLD and how to proceed with it. Once made publicly available, BOLD will drastically reduce the cost of recruiting and managing study participants for researchers. Researchers will be able to reach participants from many districts around Japan, mitigating concerns about selection bias. This will benefit young PIs with limited resources and other researchers who need to lower the cost of conducting developmental research.

## Baby’s Online Live Database

Below, we describe the grand concept and implementation of BOLD. BOLD is comprised of two main systems: participant management and study management. As the core of BOLD, we adopted the cloud-based research and participant solution system provided by Sona Systems. Sona Systems provides an online/paperless system for participant/research management, and the system has been introduced at over 1,000 universities worldwide. We modified the system’s fundamental functions to increase its suitability for developmental science research. The website BOLD, powered by Sona system, can be accessed *via* https://doshisha-akachan.sona-systems.com/. The JSBS working group will direct BOLD. JSBS has been in existence for more than 20years and is financially stable, making it a suitable candidate for sustainable management of BOLD. The Center for Baby Science at Doshisha University will manage the actual administrative work and financial support. We are currently preparing to incorporate the society in the future. Because JSBS is responsible for BOLD, additions or deletions from its working group members does not affect the management of BOLD.

### Participant Management System

The participant management system is responsible for managing participant information and reward payments. Caregivers can make an account and register their personal information, including their child’s age in months. Once an account has been created, a participant can apply to (or be invited to) studies through a webpage (or via emails). After participation, reward points that are monetizable are added to the participant’s account.

### Study Management System

The study management system manages study registration and participant recruitment.

First, a prospective BOLD researcher must apply; then, two or three JSBS working group members will blindly review the application and make a report. Based on the report, the working group will choose the application that they believe will contribute to BOLD’s development. After acceptance, researchers can register their studies within BOLD; they must specify the desired attributes of participants (e.g., gender and age), what data they want to collect, and the schedule of data collection. The ethical committee review approval period and number should also be registered. Regarding ethical considerations, researchers must undergo an ethics review at their institution when they plan to use BOLD. At that time, the application for data reuse will be included in advance and will provide a legitimate basis for data sharing. When researchers run an experiment with the assistance of BOLD, they will be asked to commit to making their data available to researchers who have undergone the same admission process.

**Figure 1:**
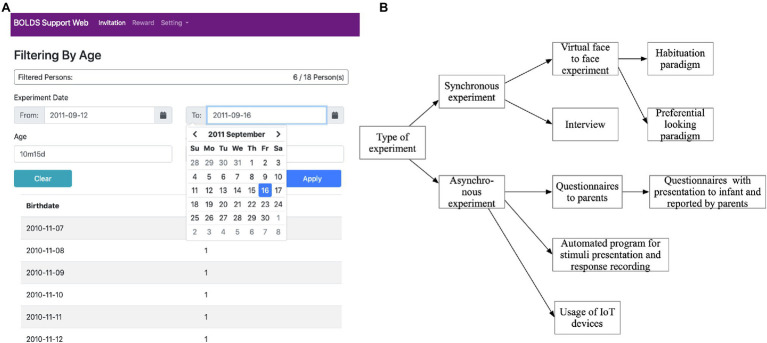
**(A)** An example of an interface for the participant management system implemented with Java language. In this screen, experimenters can select target babies/children based on the scheduled experiment dates and expected participants’ age at the scheduled dates. **(B)** Type of experiment to be integrated with Baby’s Online Live Database (BOLD).

Based on the registered information, the study management system extracts qualified participants from the participant management system. Information about the study is delivered to the qualified participants on their BOLD page and in their email. The interface for participant recruitment is shown in Figure 1A.

Potential participants receive an invitation email which directs them to the study’s page. If they decide to participate, they are prompted to click on the consent button to indicate consent for participation. This can be considered informed consent and is obtained in a digital form. The FDA allows digital informed consent (United States Food and Drug Administration, 2016). Though initiatives on digital informed consent have begun in Japan (Kogetsu and Kato, 2019), handwritten consent is still customary and therefore is accepted on BOLD. A reminder email will be sent automatically to participants just prior to the scheduled event.

Throughout all stages of data collection, personal information is confined within BOLD. Researchers are provided with only a participant ID, through which they can contact participants. It is possible for researchers to acquire personal information during experiments, such as virtual face-to-face experiments and interviews. However, these are the same as typical, offline experiments. BOLD will not limit the contents of experiments, and researchers must be responsible for the content of their experiments. It is the ethical committee’s role to protect participants’ personal information collected during experiments.

## Conducting Studies Through Bold

An advantage of BOLD is performing longitudinal studies with previously collected data. If previous study participants remain registered in BOLD, researchers can (i) collect new data from the participants and (ii) access participant data from past studies. By linking these datasets, researchers can perform a longitudinal analysis. In addition, basic cognitive, motor, and social developmental performance (the essential dataset) is collected from all participants on enrollment. This will be beneficial in longitudinal studies as the data from the initial time point are already collected.

To collect new data, BOLD navigates potential participants to websites where researchers set up their experiments and surveys. This gives researchers flexibility to conduct various types of studies using any libraries of their choice. The studies conducted are often categorized into two types: synchronous study, where participants and researchers coordinate their time and meet face-to-face over videoconferencing, and asynchronous study, where participants can participate in the survey at a time which is convenient for them, as shown in Figure 1B.

In the synchronous study, participants and researchers meet face-to-face *via* a videoconferencing system. Researchers can therefore carry out experiments and surveys, just as in the laboratory. For example, researchers can video-record infants’ faces while presenting stimuli and analyze recorded videos offline to quantify a rough estimate of fixation duration. Conventional paradigms of preferential looking and habituation–dishabituation paradigms can also be implemented online.

In the asynchronous study, three main types of studies are feasible. The first is a web-based questionnaire, in which participants answer online questionnaires at a convenient time. Some online survey systems, such as Qualtrics and SurveyMonkey, offer multimedia content presentations. Thus, it is also possible to present movies and collect responses to them. When collecting young children’s responses to multimedia content, caretakers can enter information about their children’s behavior in the survey form (e.g., Meng et al., 2021). The second method is a pre-programmed study. In this type of study, when a participant accesses a website for research, they are automatically given instructions. Participants follow the instructions and create responses that are stored in the experiment program’s server. This type of study is suitable for measuring the behavioral responses of older children and caregivers. It could also be possible to collect eye movement data using libraries such as webgazer.js. However, the validity of web-camera-based eye tracking has only been tested in adult participants, with a few exceptions (e.g., Semmelmann et al., 2017). The third method involves data collection using handy Internet-of-Things (IoT) devices (e.g., the ferro-electret sensor provided by Emfit Ltd. in Finland, used for measuring ballistocardiogram during sleep). The data collected by IoT devices can be transmitted directly to cloud servers and retrieved by researchers. Although the measurement of physiological data using IoT devices remains challenging, the results of such attempts would be significant because raw physiological data contain vast amounts of information that can be analyzed by various methods, per the researchers’ choice.

## Current Status of Bold

The implementation of BOLD is still in progress; however, the early registration of participants has already begun. The Center for Baby Science at Doshisha University began registering potential study participants in spring 2020. Registrations were made from different areas in Japan, meaning that researchers can reach people in remote areas and people who are nearby but are not able to travel to the study site due to disability. A trial recruitment period began in May 2020, and registrations rapidly increased, reaching approximately 400 over 50days. The key to BOLD’s success is creating as large a pool of potential participants as possible. To collect essential minimum data in August 2021, we will soon implement a questionnaire survey for 10-month-old infants on physical and psychological development using the Kinder Infant Development Scale (Hashimoto, 2013). We have set 10months as the minimum age due to the limitations of our research resources (i.e., we have no experts in early human development as members). We hope to lower this in the future. At present, we would like to begin with typically developing children because diagnostic information about diseases is considered personal information that requires special attention.

## Participant-Oriented Platform

Many caregivers are concerned about whether their parenting style is appropriate. However, we cannot say with confidence that developmental scientists have fully answered their questions and concerns. One way to attract potential participants is making sure that participants’ concerns are addressed and their interests are satisfied by joining the platform. It would be effective if participant can make a question to other participants as a participant-driven survey. Another way is by setting up a forum. Many of the concerns caregivers have are individualized and specific and are therefore not likely to be researched. Thus, many caregivers may want to ask caregivers with older children what to do about these problems. This can be achieved by creating a place where participants can raise questions and have them answered. Alternatively, if studies are conducted through BOLD that address caregivers’ concerns, it might increase their motivation to register.

Considering this, we carried out a preliminary survey to clarify the topics caregivers are most interested in as reference information for determining the first batch of studies to conduct using BOLD. The total number of participants was 587. The detailed procedure of the preliminary survey and the questionnaire items are described in Supplementary Material.

**Figure 2:**
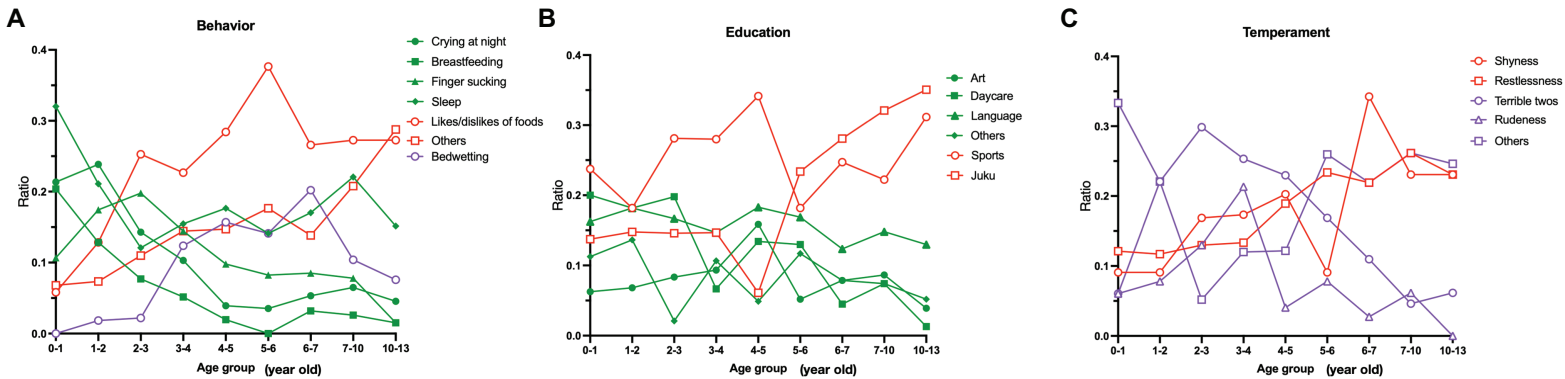
Age-dependent change of most interesting topics regarding **(A)** child’s problematic behavior, **(B)** child’s education, and **(C)** child’s temperament. The vertical axis represents the proportion of respondents who chose the topic out of all respondents in each age-group.

The main results of the first questionnaire block are presented in Figure 2. Regarding the most concerning problematic behavior (Figure 2A), frequency of choosing the “other” option increases with child’s age, indicating diversification of problematic behaviors. Figure 2B shows that caregivers have a strong interest in what kind of sports activity is most beneficial for children from the early stages of development. At around 4–5years old, interest in lessons in “Juku,” a private tutoring program for school entrance examination, steeply increases. Among topics related to children’s temperament, concerns about shyness and restlessness increase with age (Figure 2C).

In the second block of the questionnaire, respondents were asked to choose the most interesting research topics from a list of academic research topics in the developmental science field. The results are summarized in Supplementary Material. Broad topics of “Mental Development” and “Brain Development” were most frequently chosen, while more specific topics like “Development of Self Control,” “Moral Development,” and “Language Development” garnered a relatively small number of votes.

## Discussion

Baby’s Online Live Database aims to solve problems that developmental scientists are currently facing. This system simplifies participant recruitment and makes it easier to reach a large pool of participants from remote areas of the country and conduct longitudinal studies on human psychological development.

A longitudinal study with a prospective design (e.g., birth cohort study) is a powerful method for understanding the mechanisms of human development. Although there are many birth cohorts (Andersen and Casas, n.d.), our system has two prominent features that make it easier for developmental scientists to conduct longitudinal data analysis. First, our system enables researchers to recruit participants and access data from past studies in which they have participated. This makes it possible for researchers to pursue their interests without needing to obtain the necessary budget to sustain a longitudinal study. Second, new researchers are welcomed to use and make novel contributions to the system. This feature differs from the management of many other birth cohorts, where the chance of joining a longitudinal study and accessing the data are restricted to members of the research groups hosting the cohort. Owing to this openness, BOLD has the potential to accumulate longitudinal data on diverse topics hitherto neglected in existing cohort studies.

The downside of our system is that it is possible for the dataset constructed in our system to become an assortment of independent datasets that are only loosely associated with each other. However, this possibility can be reduced by collecting essential minimum dataset of great interest to many developmental scientists from all participants. We are currently deciding on the types of data to include in the essential minimum dataset, and a physical and psychological development scale (Hashimoto, 2013) should be included in the essential minimum dataset. Performance of popular behavioral tasks, such as delayed gratification tests and preferential looking to social stimuli, is also a good candidate. The inclusion of the essential minimum dataset is beneficial for both researchers and participants. It would be good motivation for researchers to use BOLD if such an attractive dataset was available. The results of the development scale included in the essential minimum dataset would also be interesting to participants. Sending reports of essential minimum datasets will increase their satisfaction.

Our preliminary questionnaire survey revealed that caregivers’ specific concerns about child development change with the child’s age. The results showed that caregivers have a strong interest in neurological and psychological development. At the same time, relatively few caregivers chose specific developmental science topics as those of most interest. These results may indicate that caregivers generally have a broad interest in children’s psychological development and that their interest is not necessarily restricted to specific cognitive functions. Non-specialists are generally unfamiliar with the recent progress of these research topics and their significance in considering children’s development. This would be one reason why these topics, though appealing to developmental psychologists, were not popular among caregivers, thus representing the gap between caregivers’ and researchers’ interests. Bridging this gap may make caregivers more willing to participate in the researcher’s study. To achieve this, researchers should increase awareness among caregivers regarding the importance of the research topics that seem at first glance irrelevant to their children’s development and, thus, uninteresting. Alternatively, BOLD may conduct a survey according to the caregivers’ interest and send them reports of the results. This gesture will make the caregivers aware that members of BOLD do care about what they truly want to know. After such experiences, caregivers may agree to participate in other more researcher-oriented studies.

Baby’s Online Live Database aims to provide solutions to the problems that developmental scientists are currently facing, primarily by reducing the cost of participant recruitment and management and simplifying the process of conducting longitudinal analyses. As the number of users increases, BOLD will become a research platform beneficial for researchers as well as participants and caregivers. It is still a small initiative, but we welcome collaborators to make it a large and international system in the future.

## Data Availability Statement

The raw data supporting the conclusions of this article will be made available by the authors, without undue reservation.

## Ethics Statement

Ethical review and approval were not required for the study on human participants in accordance with the local legislation and institutional requirements. Written informed consent for participation was not required for this study in accordance with the national legislation and the institutional requirements.

## Author Contributions

MK conceived the idea. MK and HD conceptualized the idea and wrote the original draft of the manuscript. MK, HD, XM, TM, SK, TO, and SI worked on implementing the platform. All authors contributed to the article and approved the submitted version.

## Funding

This work was supported by MEXT Promotion of Distinctive Joint Research Center Program (Grant Number: JPMXP0619217850) implemented at Doshisha University Center for Baby Science.

## Conflict of Interest

The authors declare that the research was conducted in the absence of any commercial or financial relationships that could be construed as a potential conflict of interest.

## Publisher’s Note

All claims expressed in this article are solely those of the authors and do not necessarily represent those of their affiliated organizations, or those of the publisher, the editors and the reviewers. Any product that may be evaluated in this article, or claim that may be made by its manufacturer, is not guaranteed or endorsed by the publisher.
