# Supplementary material for: Baby’s Online Live Database: An Open Platform for Developmental Science
Source: Front Psychol. 2021 Oct 13;12:729302. doi: 10.3389/fpsyg.2021.729302 (PMC8548418; doi:10.3389/fpsyg.2021.729302)
Supplement: Supplementary file 1 [file Data_Sheet_1.docx]

Supplementary Material

# Supplementary Material: Protocol and Results of Preliminary Survey

## Method

In the preliminary survey, we collected questionnaire data from parents with children aged 0 to 12 years old. The number of respondents was adjusted according to the age of their children (i.e., 65 children for each year group from 0 to 6 years, and 20 children for each year group from 7 to 12 years). The final number of respondents was 583. The survey and participant recruitment were outsourced to Cross Marketing, Inc. Since we collected a wide range of participants from all over Japan and the number of participants was not small, we believe that we have at least been able to depict the average caregiver in Japan.

The questionnaire consisted of two blocks. In the first block, respondents answered questions about their primary parenting concerns, and in the second block, they chose the academic research topics they were most interested in. The English translation of the questionnaire and answer sheet is appended below.

## Results and Discussion

Of the respondents, 73.4% were primarily responsible for childcare at the time of the survey. Out of the total number of participants, there were 367 women and 216 men, and the mean age was 38.45 years, with a standard deviation of 5.79 years. The results of the first block are presented and discussed in the main text of this paper. The table below summarizes the results of the second block.

The overall results indicated that parents are more interested in mental than physical aspects of development. At the same time, a relatively small number of parents chose specific mental development topics, such as self-control, morality, and language development.

# Questionnaire and Answer Sheet

*Preface*

We are planning to start a new research and outreach project. In this project, we will carry out online surveys about questions that mothers and fathers have about parenting and child development, such as “the cause of surprising and interesting behaviors of children” and “concerns and problems in parenting.” The final goal is to use scientific methods and statistics to answer these questions for parents.

To prepare for the project, we would like to know what you, mothers and fathers, are most interested in about child development and parenting. We would be grateful if you kindly participated in our project by completing the questionnaire below.

The first block

The first question focused on problems related to children’s behaviors. Respondents were asked to select all that applied.

Please choose this option if you are concerned about your child's sleep.

Please choose this option if you are concerned about your child's crying at night.

Please choose this option if you are concerned about your child's breastfeeding and weaning.

Please choose this option if you are concerned about your child's finger sucking.

Please choose this option if you are concerned about your child's likes/dislikes of food.

Please choose this option if you are concerned about your child's bedwetting.

Please choose this option if you are concerned about something about your child that isn’t listed above.

The second question focused on problems related to children's education/learning. Respondents were asked to select all that applied.

1. Please choose this option if you are concerned about your child's sports-related lessons.
2. Please choose this option if you are concerned about your child's art-related lessons.
3. Please choose this option if you are concerned about your child's juku.
4. Please choose this option if you are concerned about your child's kindergarten or school options.
5. Please choose this option if you are concerned about your child's language education.
6. Please choose this option if you are concerned about something other than the above that relates to your child’s education or learning.

The third question focused on problems in terms of children's temperament and personality. Respondents were asked to select all that applied.

1. Please choose this option if you are concerned about your child's rudeness.
2. Please choose this option if you are concerned about your child's shyness.
3. Please choose this option if you are concerned about your child's restlessness.
4. Please choose this option if you are concerned about your child's terrible twos.
5. Please choose this option if you are concerned about your something other than the above that relates to your child’s personality or temperament.

The second block

Please choose five items below in order of interest.

[1] Development of self-control

[2] Development of the ability to manage social relationships

[3] Physical development

[4] Development of play

[5] Risks for developmental disorders

[6] Development of communication

[7] Parent’s sleep

[8] Brain development

[9] Language development

[10] Parenting styles.

[11] Genetic influence

[12] Child’s sleep

[13] Parenting concerns

[14] Parent-child relationship

[15] Mental development

[16] Moral development

[17] Environmental influence

Table S1. The frequency with which each research topic was chosen as the most interesting

| Topics of parent's interests |  | Order of interests | | | | | | | | |
| --- | --- | --- | --- | --- | --- | --- | --- | --- | --- | --- |
|  |  | First |  | Second |  | Third |  | Fourth |  | Fifth |
| Mental development |  | 82 |  | 74 |  | 84 |  | 52 |  | 36 |
| Brain development |  | 93 |  | 69 |  | 41 |  | 40 |  | 29 |
| Physical development |  | 30 |  | 74 |  | 61 |  | 40 |  | 30 |
| Parenting concerns |  | 50 |  | 45 |  | 42 |  | 17 |  | 31 |
| Development of child’s communication |  | 21 |  | 44 |  | 55 |  | 54 |  | 52 |
| Parenting style |  | 51 |  | 46 |  | 24 |  | 23 |  | 19 |
| Parent-child relationship |  | 34 |  | 34 |  | 32 |  | 61 |  | 33 |
| Development of the ability to manage social relationships |  | 33 |  | 32 |  | 38 |  | 37 |  | 68 |
| Child’s sleep |  | 48 |  | 24 |  | 23 |  | 18 |  | 42 |
| Environmental influence |  | 20 |  | 27 |  | 41 |  | 39 |  | 31 |
| Parent’s sleep |  | 48 |  | 13 |  | 13 |  | 4 |  | 14 |
| Development of self-control |  | 15 |  | 19 |  | 21 |  | 46 |  | 46 |
| Moral development |  | 6 |  | 18 |  | 31 |  | 36 |  | 33 |
| Genetical influence |  | 17 |  | 19 |  | 20 |  | 23 |  | 24 |
| Risks for developmental disorders |  | 9 |  | 15 |  | 26 |  | 42 |  | 22 |
| Language development |  | 17 |  | 14 |  | 15 |  | 32 |  | 34 |
| Development of child’s play |  | 9 |  | 16 |  | 16 |  | 19 |  | 39 |
